# Multiomic Integration Reveals Novel miRNA–mRNA–Protein Expression Profile in the Aged Female Retina

**DOI:** 10.1167/iovs.67.11.37

**Published:** 2026-09-17

**Authors:** Nicholas W. Bariesheff, Yvette Wooff, Daniel G. Blackmore, Adrian V. Cioanca

**Affiliations:** 1 The John Curtin School of Medical Research, The Australian National University, Canberra, ACT, Australia; 2 The School of Medicine and Psychology, The Australian National University, Canberra, ACT, Australia; 3 The Shine-Dalgarno Centre for RNA Innovation, The John Curtin School of Medical Research, The Australian National University, Canberra, ACT, Australia; 4 Queensland Brain Institute, The University of Queensland, St Lucia, QLD, Australia; 5 Clem Jones Centre for Ageing and Dementia Research, Queensland Brain Institute, The University of Queensland, St Lucia, QLD, Australia

**Keywords:** aged animal model, retinal degeneration, microRNA (miRNA), miRNA–mRNA interactions, protein, inflammation, neurodegeneration

## Abstract

**Purpose:**

Aging is a leading risk factor for retinal degeneration. MicroRNAs (miRNAs) regulate posttranscriptional gene suppressors and influence inflammation and oxidative stress, two processes disrupted during retinal aging. This study aimed to identify age-related miRNA–mRNA–protein associations between young and older retinas and uncover dysregulated pathways that may contribute to retinal degeneration.

**Methods:**

Retinal function was assessed using electroretinography (ERG), and microgliosis was quantified by microglial immunohistochemistry (IHC). A multiomics approach was used to examine molecular changes in older (30-month-old) female C57BL/6J mouse retinas and compared with young female (3-month-old) controls. Illumina sequencing profiled short miRNAs (20 bp) and bulk mRNAs (150 bp), while total proteomics via mass spectrometry assessed protein expression. Bioinformatic analyses included targetome analysis (miRNet), pathway enrichment (Gene Ontology), and clustering to identify age-associated molecular targets and pathways.

**Results:**

Retinas from older mice displayed neuronal dysfunction and increased microgliosis. Sequencing revealed significant dysregulation of miRNAs linked to immune and inflammatory pathways, supported by enrichment of their predicted mRNA targets. In the older mice, mRNA expression showed broad inflammatory activation, though only 14% of dysregulated mRNAs overlapped with predicted miRNA targets. Proteomic profiling revealed a disconnect between RNA and protein expression, yet all omics layers showed enrichment in inflammatory pathways. Integrated analysis identified associations involving several gene regulatory networks in the older retina.

**Conclusions:**

This study demonstrates that at an advanced age, miRNA expression and their predicted downstream regulatory networks are dysregulated, highlighting potential molecular mechanisms underlying age-related retinal degeneration.

Aging is the leading risk factor in the development of retinal degenerative diseases, with more than half of all individuals over 60 affected by some form of retinal degeneration.^1^ Aging in itself is accompanied by several pathogenic events, including increased retinal inflammation, oxidative stress, and neuronal cell death^2^^–^^6^—hallmark features contributing to disease onset and progression.

The retina exists within an immune-privileged microenvironment, where retinal glia, comprising microglia and Müller glia, locally coordinate defense mechanisms. When photoreceptors undergo stress or death, they release signaling molecules that activate microglia, prompting their migration to the area of damage. Activated microglia in turn release proinflammatory cytokines and initiate phagocytic pathways, which further exacerbate photoreceptor degeneration. Given the direct relationship between microglia activation and photoreceptor cell death, inhibiting microglial cell activation and inflammation has been shown to improve photoreceptor survival in retinal degeneration models.^7^^,^^8^ Increased retinal inflammation and degeneration have been demonstrated in older animal models previously,^9^ but while these age-related changes have been well-characterized at the histologic level, the underlying shifts in the transcriptomic landscape, particularly within the noncoding RNA transcriptome, remain largely unexplored.

Recent studies have reported broad mRNA and long noncoding RNA expression changes in older animals^10^ and have even extended to human age-related macular degeneration (AMD) retinas.^11^^,^^12^ Analysis of both human older and AMD retinas indicates that inflammation and the immune response are among the top enriched pathways shared between aging and retinal degeneration.^11^ Despite these reports, the microRNA (miRNA) transcriptome of the older retina and its role in driving pathogenic changes such as inflammation remain unexplored.

MiRNAs possess the capacity to silence large posttranscriptional gene networks and are currently viewed as the next generation of RNA therapeutics for treating retinal degenerations,^13^^–^^16^ given their unique ability to suppress multiple biological pathways, such as inflammation. Importantly, while miRNAs suppress pathologic gene expression through targeted silencing, their absence or downregulation can have the opposite effect, leading to the activation of their biological targets, including proinflammatory mediators, oxidative stress regulators, and other contributors to neurodegeneration. Thus, miRNAs act not only as gene silencers but also as critical buffers that help maintain homeostatic control over inflammatory and degenerative signaling.

In models of acute retinal degeneration, miRNAs are known to regulate inflammation and oxidative stress. For example, miR-124, miR-155, and miR-146 expression is perturbed in the photo-oxidative damage model of retinal degeneration,^17^ and their molecular function has been linked to the activation of retinal Müller glia, polarization of retinal microglia, secretion of proinflammatory cytokines, and trafficking of circulating monocytes into the degenerating retina.^17^^–^^21^ Although miRNAs have been shown to effectively modulate inflammation in various disease models, it remains unclear how their expression is altered during normal aging and whether such changes actively contribute to the molecular mechanisms underlying age-related retinal dysfunction. Notably, most studies investigating RNA involvement in retinal disease focus on transcript-level changes, with limited integration of proteomic data. This is particularly true in the context of aging, where the downstream impact of miRNA dysregulation on RNA expression and then on protein expression—arguably the most functionally relevant outcome—remains largely unexplored. The absence of comprehensive proteomic profiling in both disease and aging models has left a critical gap in understanding how age-associated changes in miRNA expression translate into functional protein-level alterations that drive retinal degeneration in aging. Here, we show numerous differentially expressed miRNAs, mRNAs, and proteins in an aged animal model that correlate with proinflammatory gene networks. Many of these molecular alterations were associated with inflammatory processes, suggesting a central role for chronic inflammation in advanced retinal age.

## Methods

### Animal Experiments

All experiments were conducted in accordance with the ARVO Statement for the Use of Animals in Ophthalmic and Vision Research and with the approval of the Australian National University's (ANU’s) Animal Experimentation Ethics Committee (Ethics Protocols: AE2023/318). Female mice were selected to minimize aggression-related confounds in our group-housed longitudinal aging study. For the older cohort, six adult female C57BL/6J wild-type (WT) mice were purchased from Ozgene Animal Resources Centre (ARC) (Canning Vale, Western Australia, Australia) and housed at the University of Queensland (St Lucia, Queensland, Australia, AE2021/000861) under 12-hour light/dark cycle conditions (5 lux) with ad libitum access to food and water until they reached age 30 months (30 mo). For the young cohort, six adult female C57BL/6J WT mice, aged 3 months (3 mo) at experimental commencement, were housed at the Australian Phenomics Facility (Australian National University) under 12-hour light/dark cycle conditions (5 lux) with ad libitum access to food and water. To minimize environmental variability, aged mice were transferred to ANU and acclimatized for 7 days prior to tissue collection under housing conditions identical to the young cohort. All mice were maintained on the same standardized diet (Specialty Feeds #SF00-105, Glen Forrest, WA, Australia) and had ad libitum access to food and water. Older mice were meticulously monitored and screened prior to experimental testing, including regular monitoring of body weight and daily scoring. In addition, gross postmortem examination was performed at necropsy, following retinal isolation, to assess for visible tumors or organ abnormalities. No animals included in the study showed evidence of observable systemic pathology.

Two sets of experiments were conducted. The first experimental set included electroretinography (ERG), optical coherence tomography (OCT), histologic analyses, miRNA sequencing, and RNA sequencing, with six animals per age group (*n* = 6 per group; *n* = 5 for young controls in the miRNA sequencing dataset, as one sample was not fully sequenced). The second experimental set comprised proteomic analyses, which utilized samples from six 3-month-old and five 30-month-old mice. One 30-month-old proteomics sample could not be collected due to a technical issue with tissue collection, resulting in a sample size of 5 for the older cohort.

Differential expression was analyzed using *voomWithQualityWeights()* and *eBayes()* (limma), incorporating both observation- and sample-level weights to account for variability and unequal group sizes. Power analysis based on empirical dispersion and library size estimates indicated >95% power to detect twofold changes at α = 0.05 with *n* = 5, exceeding the minimum sample size (∼4) required for 80% power, confirming that the slight imbalance did not compromise statistical robustness. See Supplementary Figures S1 and S3.

All older animals were 29.9 ± 0.1 months old, and young animals were 3.0 ± 0.1 months old at the time of functional assessment and subsequent tissue collection.

### Retinal Function Assessment

To assess retinal function, full-field scotopic (dark-adapted) electroretinography (ERG) was performed as previously described.^22^ Briefly, mice were dark-adapted overnight before ERG analysis. On the day of analysis, mice were anesthetized via intraperitoneal injection of ketamine (100 mg/kg; Troy Laboratories, Glendenning, NSW, Australia) and xylazine (10 mg/kg; Troy Laboratories). Both pupils were dilated with one drop each of 2.5% w/v phenylephrine hydrochloride and 1% w/v tropicamide (Bausch and Lomb, Rochester, New York, USA). At least 10 minutes following pupil dilation, anesthetized and pupil-dilated mice were placed on the thermally regulated stage of the Celeris ERG system (Diagnosys LLC, Lowell, Massachusetts, USA). The Celeris ERG system has combined Ag/AgCl electrode-stimulator eye probes that measure responses from both eyes simultaneously and uses 32-bit ultra-low-noise amplifiers fitted with impedance testing. Eye probes were cleaned with 70% ethanol and then a 0.3% hypromellose/0.1% dextran 70 eye drop solution (Poly-Tears; Alcon, Tuas, Singapore). The probes were then placed covering and just touching the surface of each eye. A single- or twin-flash paradigm was used to elicit a mixed response from rods and cones. Flash stimuli for mixed responses were provided using a 6500K white flash luminance range over stimulus intensities from −0.01 – 40 log⋅cd⋅s/m^2^. Raw ERG traces and oscillatory potentials were recorded and analyzed using Espion V6 Software (Diagnosys LLC). Long-exposure ERG was also used to detect RPE activity (150 cd⋅s/m^2^ for 100 ms). Data from the left eye and right eye were averaged prior to conducting statistical testing. Statistics were performed in Prism version 7.0 (GraphPad Software, La Jolla, CA, USA) using a two-way analysis of variance (ANOVA) with multiple comparisons to test for differences in a-wave and b-wave peak amplitude. Mann–Whitney *U* tests were used to compare the c-wave delay, maximum amplitude, and voltage recovery. Oscillatory potentials were analyzed using the Mann–Whitney test to compare peak amplitudes and frequency. Data were expressed as the average of both eyes and presented as mean values ± SEM.

### Optical Coherence Tomography

Following ERG, mice were transferred to a lit room for OCT analysis. Cross-sectional images of live mouse retinas were taken using a MICRON IV device (Phoenix-Micron, Bend, Oregon, USA). Cross-sectional images were taken at 1-mm increments from the optic nerve. Eye gel (GenTeal; Novartis, Fort Worth, Texas, USA) was administered to both eyes for recovery and to prevent cataracts. Using OCT cross-sectional retinal images and ImageJ V2.0 software (National Institutes of Health, Bethesda, MD, USA), the thicknesses of the outer nuclear layer (ONL) and inner nuclear layer (INL) for both eyes were quantified at five different locations per cross section. Retinal layer thicknesses from both eyes were averaged and presented as the average of the two measurements.

### Retinal Tissue Collection and Preparation

Following ERG/OCT analysis, mice were euthanized via cervical dislocation. The superior portion of the left eye was marked and enucleated using curved forceps and scissors under a microscope. A small amount of 4% paraformaldehyde (PFA) was injected into the eye through the limbus before the eye was immediately immersed in 4% PFA for 3 hours. Eyes were then cryopreserved in 30% sucrose solution overnight and cryosectioned at 12 µm in a parasagittal plane (superior to inferior) using a CM 1850 Cryostat (Leica Biosystems, Nußloch, Germany). To ensure consistent comparisons were made for histologic analysis, two sequential sections at the optic nerve location per eye were used for analysis. For RNA and protein analysis, the retina from the right eye was excised via a corneal incision using an ophthalmic blade and carefully separated from the lens and RPE to minimize contamination, then placed into RNA lysis (Thermo Fisher Scientific) or RIPA buffer solution (cat. 89900 [Thermo Fisher], prepared 10 mL stock solution mixed with 1 complete ULTRA Tablets, Mini, EDTA-free, EASYpack Protease Inhibitor Cocktail, cat. 5892791001 [Sigma-Aldrich, St. Louis, MO, USA]), and stored at −80°C until further use. See the RNA and protein sections below for details of extraction.

### Histology and Immunostaining

#### Hematoxylin and Eosin Staining

Retinal cryosections were stained with hematoxylin for 2 minutes and counterstained with eosin for 3 minutes, then washed twice in PBS.

#### Immunohistochemistry

Immunohistochemical analysis of retinal cryosections was performed as previously described.^23^ Sections were immunolabeled for IBA-1 (1:500, anti-Iba1, Rat, ab283346; abcam, Cambridge, England, UK) to identify retinal microglia and macrophages, and cell nuclei were visualized using the DNA-specific dye bisbenzimide (1:10,000; Sigma-Aldrich). IBA-1^+^ cells were quantified across the entire retina and normalized to the total retinal area, averaged across two consecutive retinal sections at the optic nerve of the left eye per mouse. Quantification was performed by two researchers blinded to the experimental groups and presented as the average of the two measurements.

### TUNEL Assay

A TUNEL assay was used as a measure of photoreceptor cell death. TUNEL in situ labeling was performed on retinal cryosections using a Tdt enzyme (cat. 3333566001; Sigma-Aldrich) and biotinylated deoxyuridine triphosphate (cat. 11093070910; Sigma-Aldrich) as previously described.^23^ The total number of TUNEL^+^ cells was counted across the entire retina and normalized to the total retinal area using two consecutive retinal sections at the optic nerve head. These quantifications were performed by two researchers blinded to the experimental groups and taken as an average of the two measurements.

### Image Acquisition and Quantification

Fluorescent images were visualized and images taken using a laser-scanning A1^+^ confocal microscope at 40× magnification (Nikon, Tokyo, Japan), controlled with NIS Elements AR software, which acquired sequential 1.0-µm z-stacks and subsequently process through maximum-intensity projection in ImageJ V2.0.^24^ As retinal sections were only 12 µm thick, the likelihood of cells in upper optical planes obscuring those in deeper planes was minimal, thereby reducing potential counting errors associated with projection-based analysis. Hematoxylin and eosin (H&E) cryosections were imaged using a bright-field microscope at the Centre for Advanced Microscopy (CAM, ANU, Canberra, ACT, Australia). Images were analyzed using ImageJ V2.0.^24^ For normalization of histologic counts, total retinal area was quantified in mm^2^. To quantify retinal health, the thicknesses of the ONL and INL on retinal H&E cryosections were quantified across four distances, both superior and inferior to the optic nerve, presented as eccentricity from the optic nerve head. Photoreceptor cell row quantification was performed five times per retina using two consecutive retinal cryosections at comparable locations per mouse by two researchers blinded to experimental groups.

### Total RNA and Small RNA Extraction and Sequencing

Retinas stored in RNA lysis buffer at –80°C were thawed, and total RNA was extracted using a mirVana Total RNA Isolation Kit (#AM1561; Thermo Fisher Scientific) according to the manufacturer's instructions and split into two aliquots (one for total RNA sequencing and one for miRNA sequencing). The concentration and purity of each RNA sample were assessed using the ND-1000 spectrophotometer (Nanodrop Technologies, Wilmington, DE, USA).  RNA integrity was evaluated prior to RNA sequencing using a 2100 Agilent Bioanalyzer (Agilent, Waldbronn, Germany; Supplementary Fig. S1), where all samples with a RNA Integrity Number (RIN) value below 8.0 were excluded from RNA sequencing. Some RIN values were not calculated due to technical issues with the instrument.

### High-Throughput Sequencing and Bioinformatics

Following RNA extraction as described above, one aliquot of total RNA was dried in stabilization tubes overnight (NovogeneAIT Genomics Singapore Pte, Singapore) and shipped to NovogeneAIT Genomics Singapore for bulk RNA sequencing. Sequencing libraries were constructed with the Illumina TruSeq (Madison, Wisconsin, USA) unstranded RNA library preparation kit using polyA selection for mRNA enrichment, then sequenced on the Illumina NovaSeq6000 platform, acquiring ∼20 million 150-bp paired-end reads per sample.

Raw reads were processed with cutadapt to trim adaptors. FastQC sequence quality analysis was performed before and after cleaning. The average clean read length was 45 nucleotides for mRNA libraries. Global high-throughput sequencing of retinal mRNAs was performed to analyze overall changes in the retina between young and older mouse retinas. In total, 40 to 70 million cleaned reads were obtained and then aligned to the mm39 mouse genome and annotated according to the Gencode comprehensive annotation, further using the RSubread and EdgeR packages^25^ for sorting and statistical analysis. Mapped reads were summarized using Feature Counts^26^ and annotated based on the genomic coordinates provided by Gencode. Trimmed mean of M-values (TMM)^27^ was used for normalization (Supplementary Fig. S1). Differential expression was analyzed using the *limma-voom* package with a linear model fit and Bayes’ adjustment of *P* values to account for type I errors and multiple comparisons. Significance was determined as adjusted *P* < 0.05 (Supplementary Table S2). Subsequent biological pathway enrichment was performed using the enrichR package in R, using Gene Ontology (GO).^28^ Reference literature for age-related gene expression changes was downloaded from Zhuang et al.^29^ Pathways associated with aging and regulated cell death were identified by keyword matching against pathway names from GO and Kyoto Encyclopedia of Genes and Genomes enrichment analyses. Aging-related pathways were defined using terms including aging, senescence, longevity, lifespan, oxidative stress, mitochondrial dysfunction, DNA damage, autophagy, FOXO, AMPK, mTOR, and insulin signaling. Cell death–related pathways were defined using terms associated with apoptosis, necroptosis, ferroptosis, pyroptosis, autophagy, oxidative stress–induced cell death, caspase signaling, mitochondrial apoptotic signaling, TNF signaling, p53 signaling, NF-κB signaling, and MAPK signaling.

For miRNA sequencing, a second aliquot of total RNA was dried in stabilization tubes overnight and sequenced at the Biomolecular Resources Facility (Australian National University, Canberra, Australia). Sequencing libraries were constructed using the D-Plex Small RNA-Seq library preparation kit (cat. C05030001) and then sequenced on the Illumina NextSeq2000 (Diagenode, Denville, NJ, USA) platform acquiring ∼100 million 100-bp single-end reads per sample. The average clean read length was 20 nt for miRNA libraries. Three to 9 million reads were obtained and aligned to the mature mouse mm10 miRNA sequences downloaded from miRBase. The miRNAs were ranked based on their mean expression level (Supplementary Table S5). The TMM method^27^ was used for normalization, and the *limma-voom* package with sample quality weights was used to detect differentially expressed genes. Hierarchical clustering was performed using the *heatmap.2* function in R. Differentially expressed miRNAs were uploaded to the miRNet database^30^ to identify predicted miRNA targets, which were used for correlation analysis.

### Total Protein Extraction and Proteomic Analysis

#### Protein Extraction

Retinas stored in RIPA buffer at −80°C were thawed and retinal protein was extracted via homogenization and sonication for 5 minutes, followed by centrifugation at 10,000 × *g* for 10 minutes. The supernatant was collected, and total protein concentration was measured using the Pierce Rapid Gold BCA Protein Assay Kit (cat. A53225; Thermo Fisher) (Supplementary Table S1).

#### Sample Preparation for Global Proteome Label-Free Quantification

The single-pot, solid-phase–enhanced sample preparation (SP3) method was utilized for global proteomics.^31^ Briefly, 50 µg of protein per sample was resuspended in 100 µL of 20 mM ammonium bicarbonate before undergoing reduction with tris(2-carboxyethyl)phosphine at a final concentration of 5 mM and incubation at 60°C for 20 minutes. Following reduction, the disulfide bonds underwent alkylation with iodoacetamide at a final concentration of 20 mM and incubation in the dark at room temperature for 10 minutes. The proteins were mixed with carboxylate-modified SpeedBeads in a 1:1 ratio (65152105050250 and 45152105050250; Cytiva, Uppsala, Sweden). The beads were separated using a magnetic rack, and the supernatant was discarded before undergoing three washes with 80% ethanol, discarding the supernatant each time. Trypsin was then added to the samples at a ratio of 1:40 (w/w enzyme/protein) in 20 mM ammonium bicarbonate and incubated at 37°C overnight at 900 rpm on the thermomixer. The peptide solution was separated from the beads using a magnetic rack and collected prior to acidification by the addition of trifluoroacetic acid to 0.5% (v/v). The peptides then underwent desalting using STAGE tips.^32^

### Liquid Chromatography/Tandem Mass Spectrometry Analysis of Peptides

The mass spectrometry acquisition method for the global proteome was performed on a Thermo Ultimate 3000 RSLCnano UHPLC system with a Thermo Fusion (Thermo Fisher, Bremen, Germany) mass spectrometer. Peptides were reconstituted in 50 µL of 0.1% formic acid. Peptides were loaded onto a PepMap C18 5-µm 1-cm trapping cartridge (Thermo Fisher Scientific) at 14 µL/min and washed for 6 minutes before switching the precolumn in line with the analytical column (nanoEase M/Z Peptide BEH C18 Column, 1.7 µm, 130 Å and 75 µm ID × 25 cm; Waters, Milford, MA, USA). The separation of peptides was performed at 250 nL/min using a linear ACN gradient of buffer A (0.1% (v/v) formic acid, 2% (v/v) ACN) and buffer B (0.1% (v/v) formic acid, 80% (v/v) ACN), starting at 5% buffer B to 35% over 65 minutes, then rising to 80% B over 15 minutes, followed by 5% B for 10 minutes. The total runtime was 90 minutes. Data were collected on a Thermo Fusion Orbitrap (Thermo Fisher Scientific) in Data Independent Acquisition mode using *m/z* 375 to 1500 as the mass spectrometry (MS) scan range, and higher-energy collisional dissociation (HCD) tandem mass spectrometry (MS/MS) spectra were collected in the Orbitrap using loop count = 20 with a loop cycle time of 3 seconds. Other parameters for the instrument were as follows: MS1 scan at 60,000 resolution, maximum injection time of 118 ms, HCD collision energy of 30%, and automatic gain control (AGC) target of 400,000. MS2 scan was at 15,000 resolution, 400 to 1000 *m/z* scan range, collision energy of 30%, AGC of 400,000, and maximum injection time of 22 ms. The isolation window of the quadrupole for the precursor was 1.4 *m/z*. Internal lock masses of 445.12003 *m/z* and 667.1764 *m/z* were used.

### Liquid Chromatography–MS/MS Data Processing and Analysis

Raw files were searched against the UniProt mouse protein database using DIA-NN 1.9, where the precursor false discovery rate was set to 1%, with carbamidomethylation of cysteine, oxidation of methionine, and N-terminal methionine excision set as fixed modifications. Since missing values were associated with lowly expressed proteins, missing values were imputed using the *impute* R package with the “MinProb” method to minimize bias from low-abundance proteins. Following imputation, the data were normalized using a variance-stabilizing transformation, and the effectiveness of the normalization procedure was verified by plotting boxplots for all samples and ensuring they all had similar medians (Supplementary Fig. S3). Differential expression was conducted using the *limma* R package,^33^ where the adjusted *P* value was set at <0.05 and the fold change (FC) was set to >0.5 or <−0.5. The resulting data were presented using GraphPad Prism (version 7.0) and Morpheus^34^ (version 8.2.1). Network analysis was performed using STRING,^35^ and overrepresentation analysis was performed using *enrichR* with GO^28^ Biological Processes 2025 as the reference set.

### Statistics and Data Analysis

Statistical analyses for ERG and histological data were performed using GraphPad Prism (version 7.0). Comparisons between two groups were conducted using Mann–Whitney *U* tests, and multiple group comparisons were performed using ANOVA with appropriate post hoc corrections. Transcriptomic, miRNA sequencing, and proteomic data were analyzed using RStudio (version 2024.12.0+467), with differential expression determined using adjusted *P* values <0.05 and an absolute log_2_FC >0.5.

### Data Availability

All raw RNA sequencing datasets supporting the conclusions of this article are available on the Sequence Read Archive (Bioproject ID PRJNA1284443) as part of the National Centre for Biotechnology Information. Raw RNA reads before normalization are available in Supplementary Figure S1. The mass spectrometry proteomics data have been deposited in the ProteomeXchange Consortium via the PRIDE partner repository^36^ with the dataset identifier PXD065636. Raw mass spectrometry reads before normalization are available in the supplementary material.

## Results

### Mice Aged 30 Months Display Impaired Visual Function, Increased Microgliosis, and Altered Retinal Morphology

**Figure 1.**
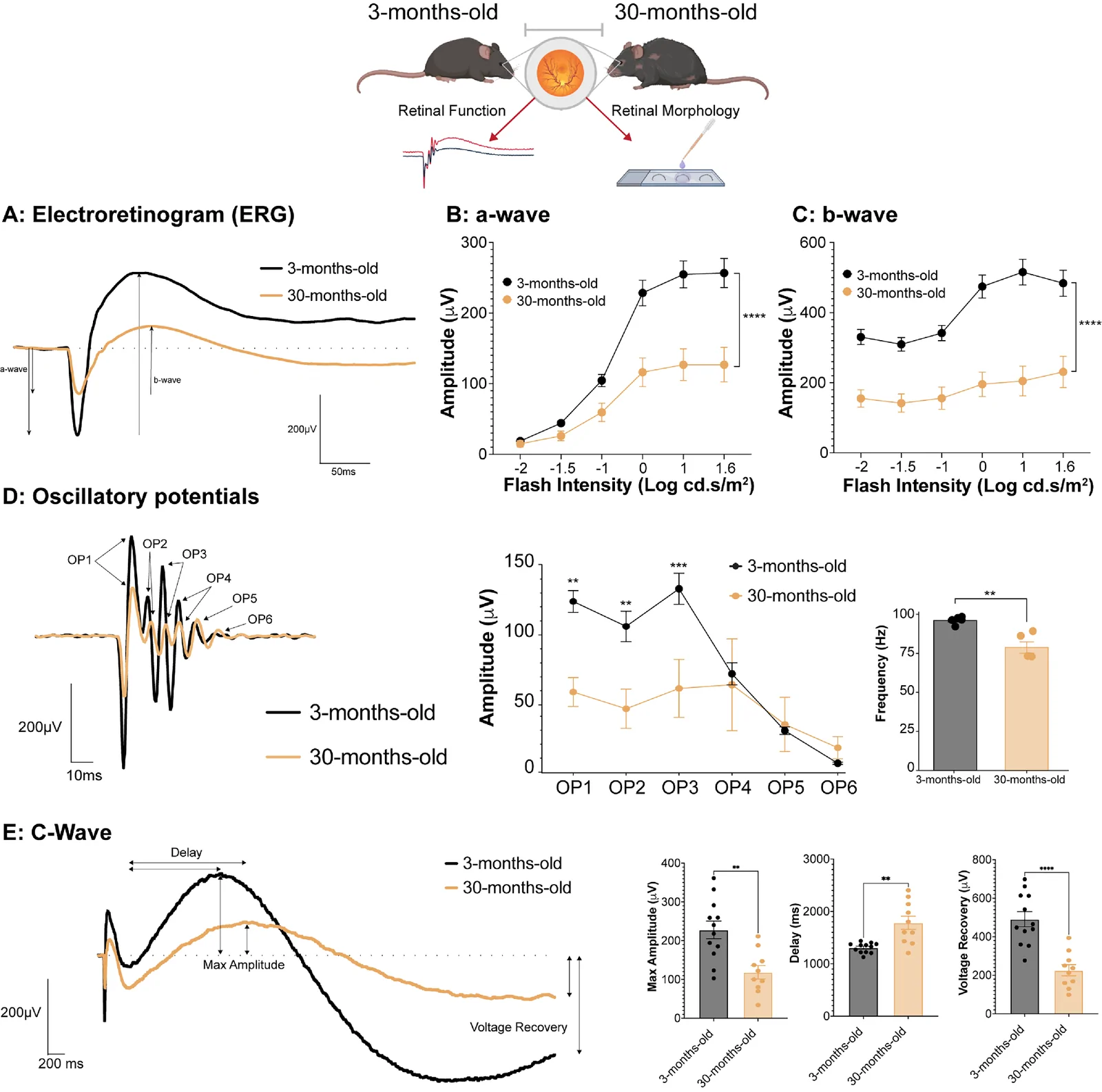
Thirty-month-old mice display impaired retinal function compared to young controls. (**A**) Retinal function was measured using scotopic ERG. Peaks of the a- and b-waves are indicated with *arrows*. (**B**) The a-wave amplitudes were significantly lower in 30-mo mice compared to 3-mo controls. (**C**) The b-wave amplitudes were significantly lower in the 30-mo mice compared to 3-mo controls. (**D**) OP raw trace shown. OP amplitudes and frequency were significantly lower in the 30-mo mice compared to 3-mo controls. (**E**) The c-wave, raw trace shown. The c-wave amplitude, delay, and voltage recovery were all significantly lower in the 30-mo mice compared to 3-mo controls. Data are represented as mean ± SEM (*n* = 6; *P < 0.05, **P < 0.01, ***P < 0.001, ****P < 0.0001).

To establish that 30-month-old mice experience retinal dysfunction with age, retinal function was evaluated using electroretinography (ERG). ERG responses under dark-adapted (scotopic) conditions were recorded in young adult (*n* = 6) (3 mo) and older (*n* = 6) (30 mo) mice (raw traces recorded at the highest flash intensity are shown in Fig. 1A). It was found that a-wave amplitude (photoreceptor response) was significantly lower in older mice by approximately 50% compared to the young controls (Fig. 1B; 3 mo: 256.7 ± 20.6 µV; 30 mo: 126.9 ± 24.5 µV; *n* = 6, *P* < 0.0001; two-way ANOVA with multiple comparisons). Similarly, b-wave amplitudes (second-order neurons/bipolar responses) in the older mice were approximately 50% lower than those seen in the young controls (Fig. 1C; 3 mo: 484.2 ± 37.1 µV; 30 mo: 231.0 ± 44.6 µV; *n* = 6, *P* < 0.0001; two-way ANOVA with multiple comparisons).

Next, oscillatory potential (OP) amplitudes were analyzed for the highest flash-intensity stimulus, reflecting overall changes in retinal interneurons. It was found that older mice had an overall lower OP frequency, with a mean of 81.1± 3.9 Hz compared to that of the young controls (97.3 ± 1.8 Hz; *n* = 6, *P* < 0.05; Mann–Whitney *U* test), and also a significantly lower amplitude for OP3 (Fig. 1D; 3 mo: 199.4 ± 11.2 µV; 30 mo: 95.0 ± 24.6 µV; *n* = 6, *P* < 0.05; Mann–Whitney *U* test), indicating interneuron dysfunction.

To evaluate RPE function, the c-wave of young and older mice was analyzed through the use of a prolonged exposure flash stimulus (150 cd⋅s/m^2^ for 100 ms). The 30-mo mice exhibited significantly lower c-wave maximum amplitude (118.6 ± 17.1 µV; *n* = 6, *P* < 0.05; Mann–Whitney *U* test), longer c-wave delay (1783 ± 128.1 ms; *n* = 6, *P* < 0.05; Mann–Whitney *U* test), and reduced c-wave voltage recovery (226.1 ± 28.9 µV; *n* = 6, *P* < 0.05; Mann–Whitney *U* test) (Fig; 1F) compared to the 3-mo controls (228.0 ± 22.8 µV, 1307 ± 27.4 ms, and 491.8 ± 38.5 µV, respectively).

These results demonstrate clear functional retinal dysfunction in the older cohort compared to the young controls, supporting 30 months as a suitable time point to represent an advanced age and the associated visual changes expected.

**Figure 2.**
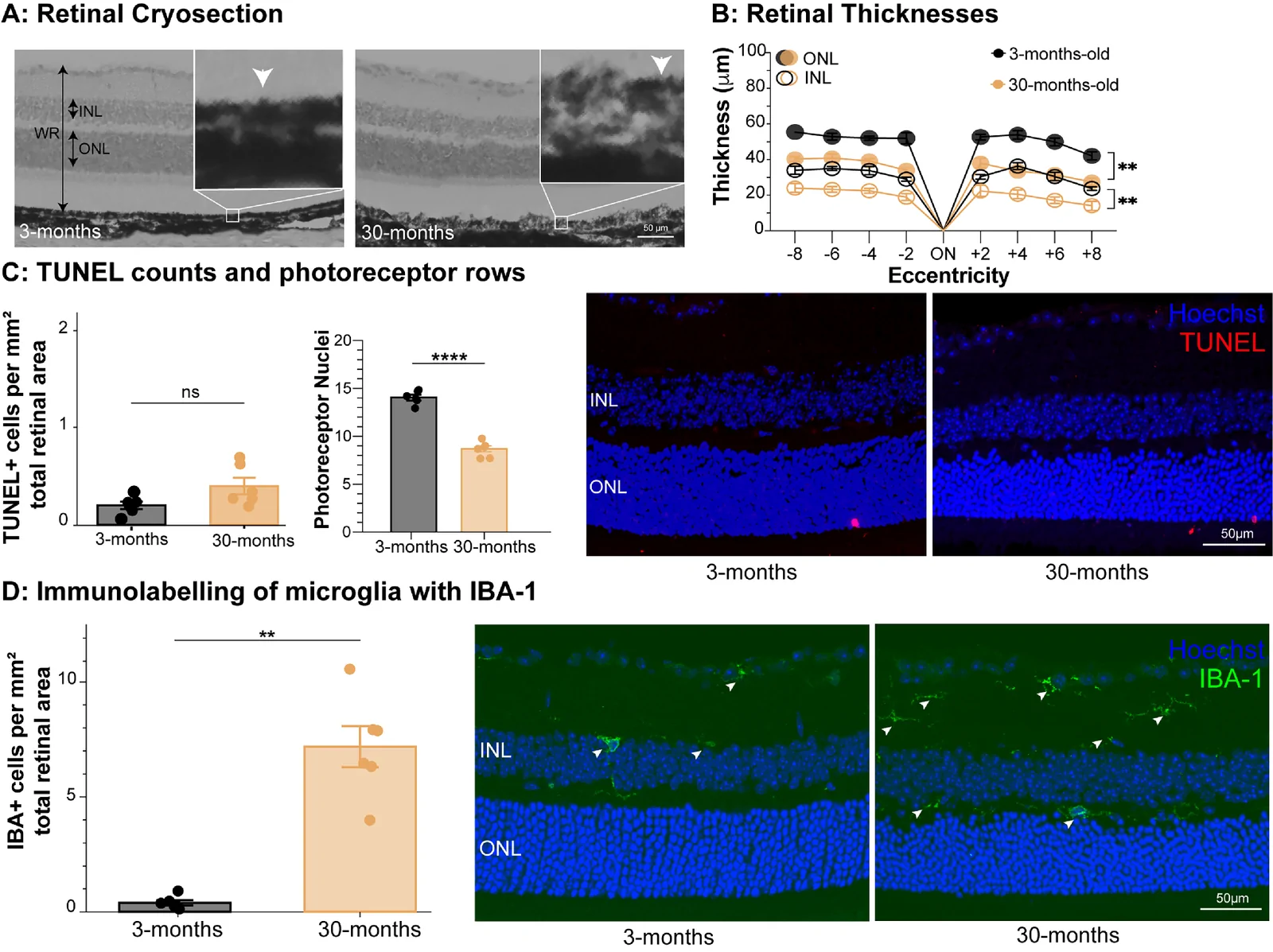
Thirty-month-old mice display impaired retinal morphology and increased microgliosis but no changes in photoreceptor cell death compared to young controls. (**A**) Retinal cryosections were stained with H&E to measure retinal thickness. *White arrowheads* indicate retinal pigmented epithelial cells. *Scale bar*: 100 µm. (**B**) ONL and INL thicknesses were each significantly lower in 30-mo mice than in 3-mo controls. (**C**) The 30-mo mice showed no changes in TUNEL-positive cells compared to 3-mo controls and also had fewer photoreceptor rows compared to 3-mo controls. (**D**) The 30-mo mice showed a higher number of IBA-1^+^ cells across the whole retina compared to 3-mo controls. Data are represented as mean ± SEM (*n* = 6; **P < 0.01, ***P < 0.001, ****P < 0.0001). *Scale bar*: 50 µm.

Next, key morphologic changes to retinal structures, indicative of degeneration, were investigated by preparing retinal cryosections. Older mice showed significantly reduced ONL and INL thicknesses (Fig. 2A), the layers in which neuronal cell bodies of photoreceptors and support cells reside. While the RPE of young mice displayed expected linearity to support overlying photoreceptors, older mice presented with no linearity in its morphology, further indicating degeneration (Fig. 2A). These morphologic changes are consistent with the observed reduced c-wave amplitude of the ERG, which reflects degeneration of the RPE.

To investigate whether these functional and morphologic degenerative changes were due to dying photoreceptor cells, a TUNEL assay was performed on retinal cryosections to label apoptotic cell bodies. Interestingly, no differences in the number of TUNEL^+^ cells were observed across the whole retina between the 30- and 3-mo control groups with a mean of 0.4 ± 0.1 total TUNEL^+^ cells per mm^2^ of total retinal area in the 30-mo group and 0.2 ± 0.04 total TUNEL^+^ cells per mm^2^ of total retinal area in the 3-mo controls (Fig. 2C; *n* = 6, *P* > 0.05, Mann–Whitney *U* test). Conversely, when photoreceptor rows were quantified in each group, it was found that the older mice presented with a mean photoreceptor number of 9 ± 0.3, compared to 14 ± 0.3 seen in the young controls (Fig. 2C; *n* = 6, *P* < 0.0001, unpaired *t*-test), indicating a lower number of overall photoreceptors, supporting the reduced ERG responses recorded (Fig. 1).

To determine whether the decline in retinal function and morphology was associated with altered levels of microgliosis, immunohistochemical labeling was performed for the microglia marker IBA-1. The older cohort presented with a significantly higher number of IBA-1^+^ cells across the entire retina, with 6.9 ± 0.9 total IBA-1^+^ cells per mm^2^ of total retinal area, compared to the young controls, which had 0.4 ± 0.1 total IBA-1^+^ per mm^2^ of total retinal area (Fig. 2D; *n* = 6, *P* < 0.01, Mann–Whitney *U* test). There were no differences between inferior and superior IBA^+^ counts, indicating that microgliosis was robust and widespread. Reduced retinal thickness was also observed in the older mice using OCT, supporting these findings and the ERG results (Supplementary Fig. S2).

These results indicated that 30-mo-old mice show robust signs of retinal dysfunction and degeneration, including loss of neuronal function, aberrant morphology, and elevated levels of microgliosis.

### Mice Retinas at 30 Months Old Present With Dysregulation of miRNAs That Could Be Associated With Inflammatory and Immune Signaling Pathways

Previous studies have shown that mRNAs are dysregulated during retinal aging.^10^^,^^11^ To unravel miRNA dysregulation associated with advanced retinal age, retinal RNA was extracted from young (3 mo) and older (30 mo) mice and sequenced for small RNAs (20 bp).

**Figure 3.**
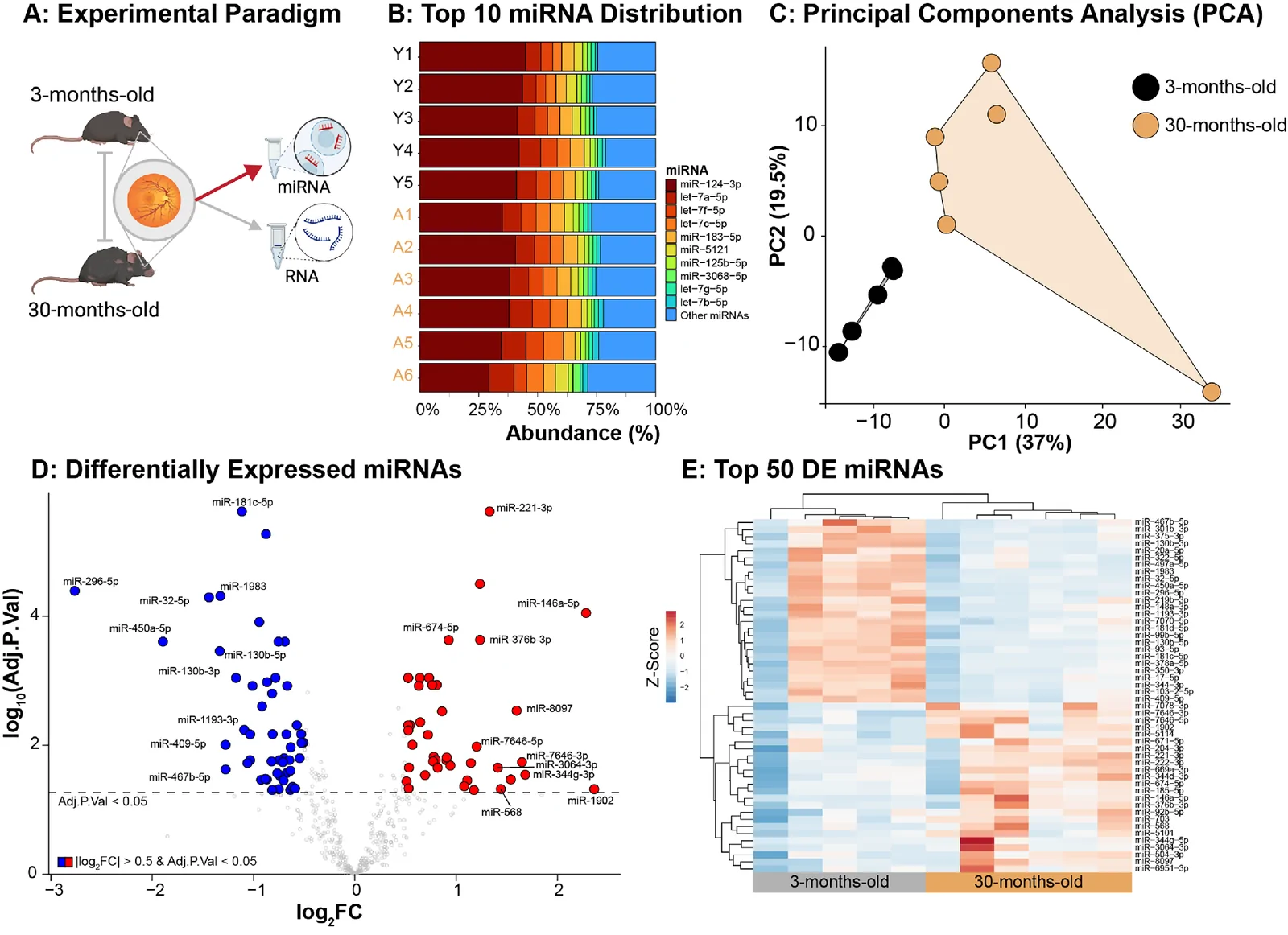
MiRNA sequencing from the retina reveals differential expression of several miRNAs in 30-month-old mice compared to young controls. (**A**) Experimental paradigm and (**B**) miRNA composition between samples based on overall expression. (**C**) Principal component analysis shows a shift to the right in the 30-mo group compared to the 3-mo control group. (**D**) Volcano plot showing the differentially expressed genes, revealing 91 differentially expressed miRNAs (49 downregulated and 42 upregulated). (**E**) Top 50 differentially expressed miRNAs listed as a heatmap. *n* = 5 for 3-mo controls and *n* = 6 for 30-mo group.

Among the most highly expressed miRNAs in both age groups were miR-124-3p, members of the let-7 family (let-7a-5p, let-7b-5p, let-7c-5p, let-7f-5p, and let-7g-5p, and photoreceptor-specific miR-183-5p (part of a cluster miR-182/96/183; Fig. 3B).^37^^,^^38^

Dimensionality reduction showed a clear separation between 3- and 30-mo transcriptomic profiles (Fig. 3C). A total of 91 differentially expressed miRNAs (adjusted *P* < 0.05 and |log_2_FC| > 0.05) were identified between 3- and 30-mo retinas (49 down, 42 up; Fig. 3D), with the top 50 shown in Fig. 3E (Supplementary Table S2).

Collectively, these findings suggest that the dysregulation of miRNAs is associated with proinflammatory gene networks in the retina at an advanced age.

### Gene Expression of mRNAs in 30-Month-Old Retinas Shows Associations With Inflammation and Immune Signaling

**Figure 4.**
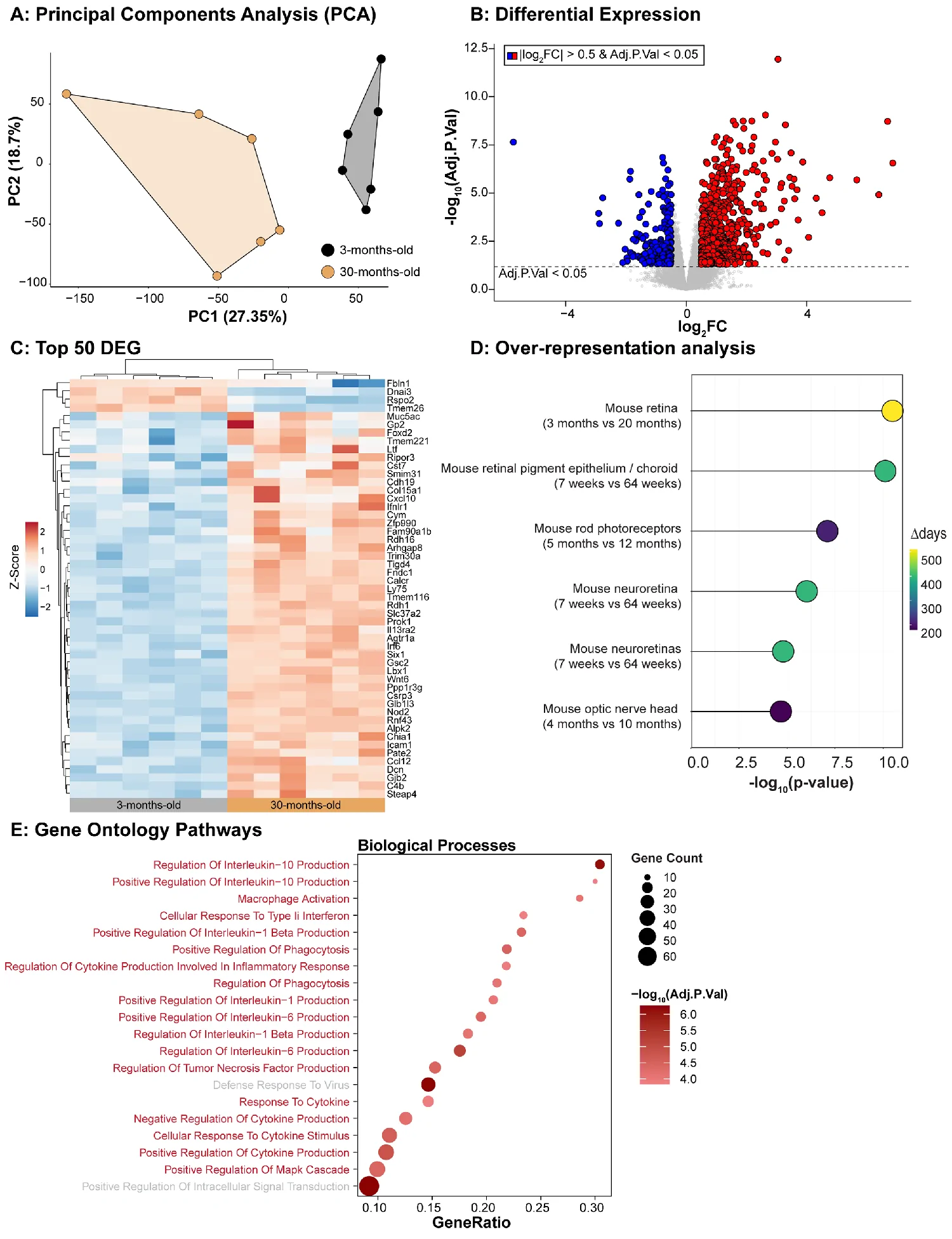
Total RNA sequencing from the retina reveals differential expression of genes in 30-month-old mice compared to young controls. (**A**) Principal component analysis shows a shift to the left in the 30-mo group compared to 3-mo controls. (**B**) Volcano plot of the differentially expressed genes reveals 756 differentially expressed genes (154 downregulated and 602 upregulated). (**C**) Hierarchal clustering of the top 50 differentially expressed genes listed as a heatmap. (**D**) Overrepresentation analysis of differentially expressed transcripts against age-related GO datasets, highlighting significantly enriched age-associated biological processes and pathways. (**E**) Biological pathway enrichment shows pathways associated with inflammation, immune signaling, and interferon signaling (*red*) to be enriched at 30 mo.

To identify potential targets of differentially expressed miRNAs, mRNA was sequenced from the same retinal samples from young (3 mo) and older (30 mo) mice retinas. A principal components analysis (PCA) was utilized to visualize the alterations in RNA profiles between 3- and 30-mo mice, which showed clear clustering and separation (Fig. 4A).

Differential gene expression analysis identified 756 mRNA transcripts to be significantly changed between 3- and 30-mo retinas (adjusted *P* < 0.05 and |log_2_FC| > 0.05; Fig. 4B) with the top 50 differentially expressed mRNAs shown in Figure 4C (Supplementary Table S3).

To determine whether these transcriptional changes are age-related, overrepresentation analysis was performed against large, independent aging reference datasets from GO, all of which include both male and female samples. Several aging-associated databases were significantly enriched within our dataset (Fig. 4D), demonstrating a strong overlap between our differentially expressed genes and previously established molecular signatures of aging.

Gene enrichment analysis through GO was used to decipher enriched pathways of these differentially expressed mRNAs. Analysis revealed several significantly enriched pathways (*P* < 0.05; Supplementary Table S4, Fig. 4E). Among the most highly enriched pathways, the inflammatory response, immune signaling, interferon signaling, apoptosis, the adaptive immune response, and multiple facets of the complement pathway were all upregulated (Fig. 4D), consistent with previous work demonstrating the key involvement of the innate immune response in the aged retina.^39^ Consistent with the lack of positive TUNEL labeling, no apoptotic or cell death–related pathways were enriched in upregulated and downregulated transcripts (Supplementary Fig. S5).

### Differentially Expressed miRNAs in 30-Month-Old Mice Retinas Control Gene Networks Involved in Glial Cell Activation and Retinal Inflammation

**Figure 5.**
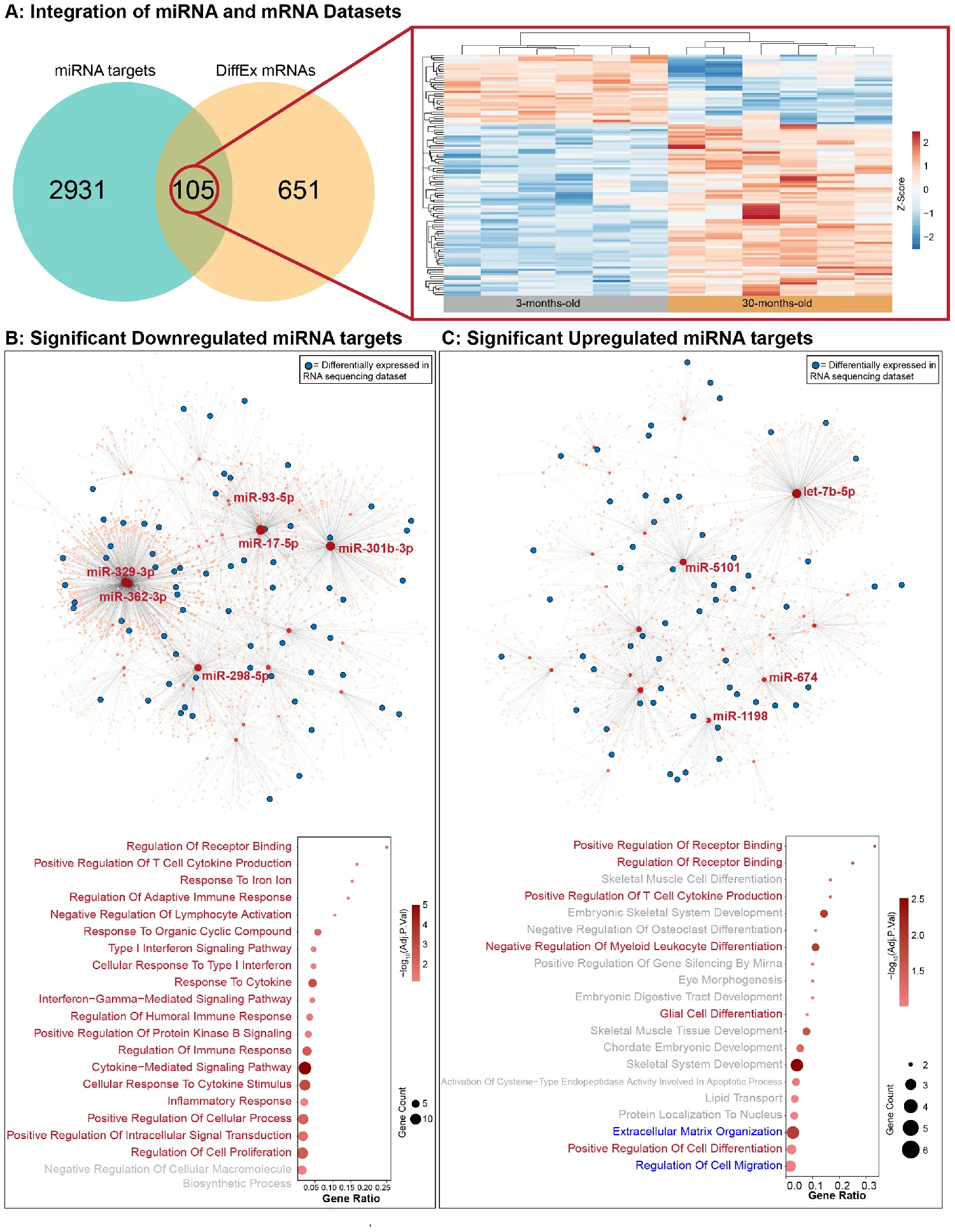
Integration of miRNA and mRNA sequencing datasets reveals distinct regulatory networks. Using the predicted mRNA targets from the miRNet database, all of the predicted targets were compared with the mRNA sequencing dataset (**A**). It was found that only 105 of the 3036 predicted mRNA transcripts were differentially expressed mRNAs. Heatmap of these 105 genes is shown, with significant intragroup clustering. These significant mRNAs are shown in the targetome of the downregulated miRNAs (**B**) and also the upregulated miRNAs (**C**). The mRNA transcripts that were differentially expressed in the RNA sequencing dataset are highlighted in *blue*. Biological pathway enrichment using GO on all these significant mRNA transcripts shows key biological pathways involved in cytokine signaling, immune response, inflammation (*red*), and extracellular matrix organization (*blue*).

To understand the potential biological function of miRNAs dysregulated in 30-month-old mice, up- and downregulated miRNAs were run through the miRNet database to get an output of their predicted mRNA targets. The miRNet database generated 3036 predicted mRNA targets, of which 105 transcripts were differentially expressed in the RNA sequencing data (Fig. 5A, Supplementary Table S5). Among the downregulated miRNAs (Fig. 5B), miR-329-3p and miR-362-3p presented the most predicted mRNA targets, with the rest being quite evenly spread. Of the 2082 predicted target mRNAs generated from the miRNet database, 64 were differentially expressed from RNA sequencing. Pathway analysis on this network was associated with biological pathways such as cytokine production, immune response, interferon signaling, and more inflammatory-related pathways (Fig. 5B). Among the upregulated miRNAs (Fig. 5C), let-7-5p made up a large percentage of mRNA interactions. Of the 1460 predicted target mRNAs generated from the miRNet database, 57 were differentially expressed from RNA sequencing. Pathway analysis on this network was associated with biological pathways such as cytokine production, glial cell organization, and extracellular matrix maintenance (Fig. 5C).

Taken together, the dysregulation of miRNAs at 30 months reveals two distinct predicted target networks associated with this advanced age in the retina. These transcripts represent biologically relevant mRNA targets likely affected as a consequence of miRNA dysregulation in the older retina.

### Total Proteomic Analysis in 30-Month-Old Retinas Reveals Inflammatory Signature Associated With Dysregulated Gene Networks

To investigate whether changes in miRNA and mRNA expression during aging translate to altered protein expression, total proteomic analysis of retinas from age-, sex-, and strain-matched young (3 mo) and older (30 mo) mice was performed using mass spectrometry. All 11 samples passed quality control (Supplementary Fig. S3) and were included in downstream analysis, leaving biological replicates of *n* = 6 for the 3-mo group and *n* = 5 for the 30-mo group.

**Figure 6.**
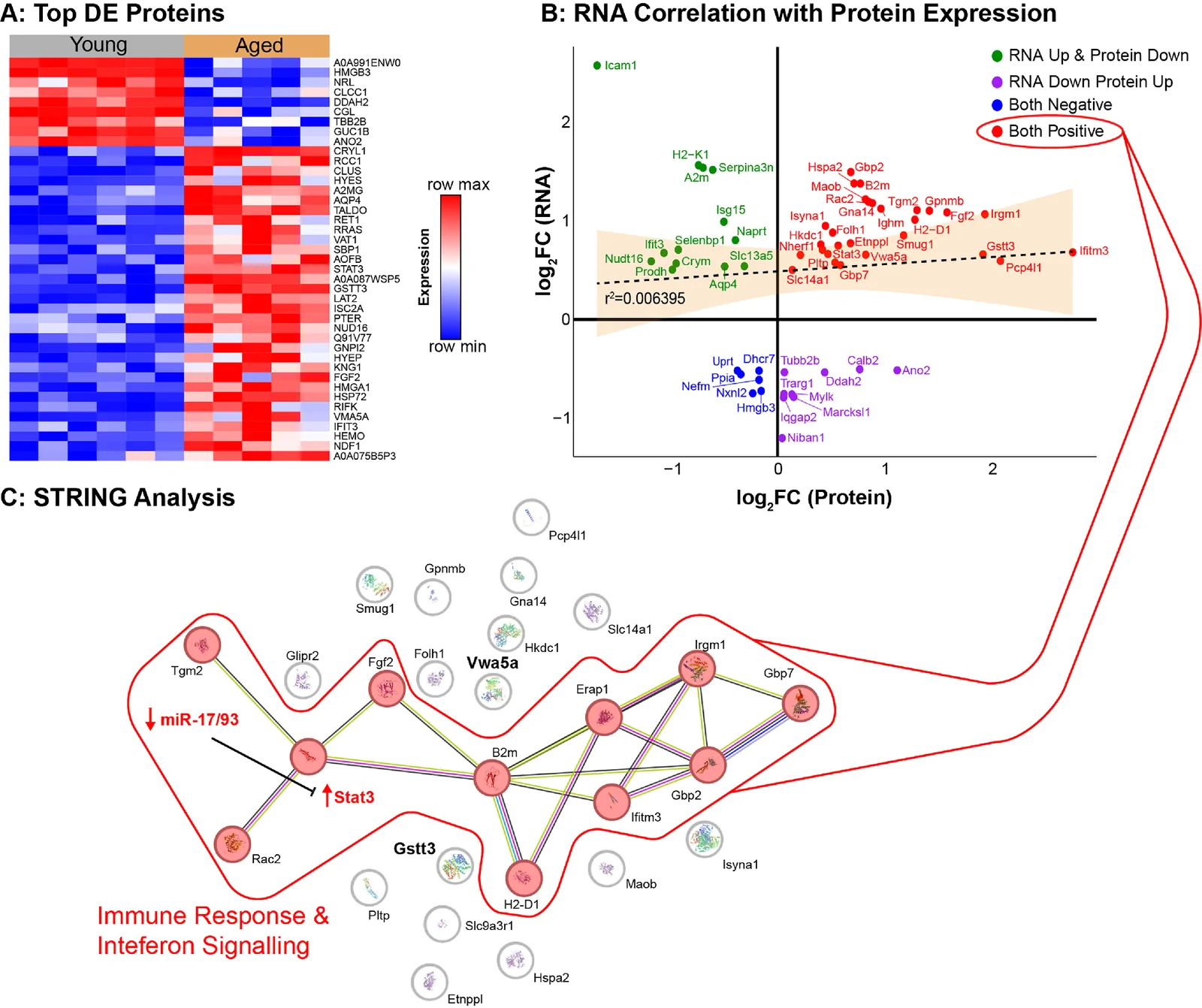
Global proteomics show dysregulation of inflammatory proteins in 30-month-old mice compared to young controls. (**A**) Top differentially expressed proteins listed as a heatmap showing strong intragroup clustering of 41 significant proteins. (**B**) Correlation of protein expression with RNA expression based on log_2_FC. (**C**) STRING analysis of the positively enriched group reveals an inflammatory protein network dysregulated at advanced retinal age, 3 of which are targets of significant miRNAs, with their corresponding mRNA transcripts also significant in the mRNA sequencing. A downregulation of miR-17/93 in 30-mo mice correlates with an increased expression of Stat3 mRNA and its protein. *n* = 6 for 3-mo controls and *n* = 5 for the 30-mo group.

A total of 41 proteins were differentially expressed between groups (adjusted *P* < 0.05; |log_2_FC| ≥ 0.5), with 32 upregulated and 9 downregulated in 30-mo retinas (Fig. 6A, Supplementary Table S6, Supplementary Fig. S4). PCA revealed clear separation of the proteomic profiles of 30- and 3-mo cohorts (Supplementary Fig. S4). Enrichment analysis of these proteins highlighted pathways including interleukin signaling and angiogenesis (Fig. 6B, Supplementary Table S7), implicating inflammation and vasculature alterations in 30-month-old mice.^40^^–^^43^

Among the most upregulated proteins were Stat3, Fgf2, Hsp72, and Kng1—all previously implicated in retinal degeneration and stress responses.^44^^–^^48^ Notably, Stat3, a known regulator of cell survival and immune responses,^49^^,^^50^ is a predicted target of miR-93, a member of the miR-17 family, which was downregulated following miRNA sequencing.

To explore correlation between transcript and protein levels, fold change values of the 43 significant proteins with their corresponding transcript from RNA sequencing were compared. Only 15 of 43 protein-coding genes were also differentially expressed at the RNA level, including Stat3.^51^ Overall, correlation between RNA and protein expression was poor (*R*^2^ = 0.006) (Fig. 6C, Supplementary Table S8), contradicting previous reports that suggests RNA expression should correlate with protein expression.^52^

Proteins could be classified into four categories based on directionality of RNA and protein expression. Of particular interest were the 28 genes and proteins upregulated at both levels, which showed strong protein–protein interactions enriched for interferon signaling and immune response pathways (Fig. 6D). These proteins represent potential targets for intervention in retinal aging.

Finally, integration of all three datasets, miRNA, RNA, and proteomics, identified three molecules consistently dysregulated across all levels: Stat3, Gstt3, and Vwa5a. Stat3 demonstrated associations as a potential inflammatory regulator and target of miR-93/miR-17, with both miRNAs also downregulated in older retinas. Gstt3, a glutathione transferase subunit, may be associated with oxidative stress in Müller and amacrine cells,^53^^,^^54^ while Vwa5a's retinal role remains underexplored.

Taken together, multiomic integration highlights novel miRNA–mRNA–protein associations in an advanced age animal model, highlighting inflammation as a key player at an advanced retinal age, which could be regulated by miRNAs at multiple levels.

## Discussion

In this study, an older animal model of retinal degeneration was used to investigate the molecular and cellular changes associated with an advanced physiologic retinal age. Older animals exhibited lower retinal function, higher levels of microgliosis, and a thinner photoreceptor layer compared with younger animals. These pathologic features were paralleled by a distinct dysregulation of miRNA expression profiles, particularly those predicted to be involved in inflammatory regulation. The altered expression of these miRNAs was reflected in the upregulation of their predicted inflammatory mRNA targets and corresponding protein products in 30-month-old animals.

In line with published work, it was demonstrated that retinal function was reduced in older animals, validating the notion that visual processing decreases with age.^55^^–^^57^ Furthermore, this reduced function correlated with reduced thicknesses of retinal layers, especially the outer nuclear layer, and also a higher number of inflammatory microglia across the retina, further supporting the idea that retinal health declines with age, parallel to an increase in retinal inflammation.^56^ No differences in the number of detectable TUNEL-positive cells were observed in the outer retina, and analysis did not reveal enrichment of apoptotic signaling pathways or differential expression of key proapoptotic markers (Supplementary Fig. S5). However, photoreceptor nuclei were significantly reduced, indicating that cell loss had occurred despite the absence of detectable apoptotic DNA fragmentation at the time of analysis. This could be explained by a slow para-inflammatory process responsible for cell debris removal, during which active cell death is not detectable. Together, these findings suggest that this aged model may represent a valuable platform for the study of para-inflammation and could be used to further understand the mechanisms underlying this process. Future studies should aim to combine this model with models of detrimental inflammation to better understand how to maintain a homeostatic environment, while also working to unravel the transcriptomic signature of the transition from para-inflammation to detrimental inflammation, providing critical molecular insight into this important biological threshold. Additionally, the proinflammatory microenvironment itself may indirectly contribute to outer nuclear layer thinning by impairing photoreceptor homeostasis, inhibiting regenerative or progenitor-like responses,^58^ or promoting synaptic remodeling^59^ and atrophy rather than acute cell death, highlighting the importance of examining earlier age time points and alternative cell death markers to better capture the active degenerative process.

To date, there have been reports of long noncoding RNA changes in the aged mouse retina,^10^ single-cell mRNA changes of the aged mouse retina,^60^ and single-cell RNA changes in the aged human retina.^61^^,^^62^ Here, for the first time, changes to small noncoding miRNA expression in the mouse retina at an advanced age are reported, with 91 miRNAs shown to be differentially expressed in the 30-month-old mouse retina. In the context of retinal disease, previous work has shown, using acute retinal degeneration models, that key miRNAs, such as miR-124^14^^,^^17^ and miR-155,^20^^,^^21^ play an important role in retinal degeneration, but no changes in the expression of these miRNAs were observed in naturally aged mice. This may occur due to the acute and dynamic nature of current models for degeneration, where they are effective in mimicking the very late stages of retinal degeneration, but are not entirely accurate at modeling true physiologic aging. It is important to acknowledge that naturally aged animals, without induced pathology or comprehensive ophthalmic phenotyping, have an undefined retinal health status. While our data do not represent a formal disease model, they provide a relevant aging context; thus, our reporting of changes in inflammatory-associated miRNAs should be interpreted within this framework rather than as disease-specific evidence. To contextualize the biological relevance of this time point, we expanded our pathway-level analyses and compared our aging signatures against independent mouse and human aging datasets. These analyses show strong concordance with transcriptional changes observed in late-life human aging, particularly comparisons approximating young-adult versus elderly individuals (approximately 20 vs. 70 years), as well as established mouse aging datasets spanning advanced age contrasts.^29^ This cross-species congruence supports the interpretation that the molecular programs we identify are representative of terminal aging biology, rather than idiosyncratic pathology arising from moribund animals.

Our data report several novel miRNAs that are dysregulated at an advanced age. Notably, a significant downregulation in the expression of miR-17-5p and miR-93-5p, both key members of the miR-17 family, was seen. This downregulation was strongly correlated with changes in their predicted mRNA targets and protein counterparts. Notably, multiomic integration identified signal transducer and activator of transcription 3 (Stat3) as one of three predicted mRNA targets associated with this dysregulation. Given Stat3’s established role in multiple neurodegenerative diseases,^63^^,^^64^ its dysregulation in the retina at an advanced age is significant, as it is a predicted target by members of the miR-17 family, namely, miR-17-5p and miR-93-5p. It has been postulated that miR-17 interacts heavily with miR-92, forming a cluster associated with aging and cancer.^65^^,^^66^ In a study by Abdelhamid et al.,^64^ it was shown that an increase in miR-17 expression interacts with Stat3 by directly targeting and inhibiting its expression in breast cancer cells. In contrast to these previous observations, our findings reveal a downregulation in miR-17 and miR-93 expression in older mice, which correlates with upregulated Stat3 expression at both the RNA and protein levels, suggesting that Stat3 upregulation may be a feature of an older retina. In the context of retinal disease, miR-17 has been reported to be elevated in the plasma of patients with AMD, while others have shown it to be downregulated in peripheral blood mononuclear cells (PBMCs) of patients with dry AMD, which may reflect tissue-specific regulation and differential release of miRNAs into circulation. In the aged retina, an observed downregulation of miR-17 may relieve repression of its inflammatory targets, such as Stat3, contributing to a proinflammatory environment characteristic of advanced retinal age, with corresponding degenerative changes. In the retina, it has been shown that miR-93/Stat3 signaling plays a key role in microglial cell activation.^45^ In an acute ocular hypertension model of glaucoma, reduced miR-93 expression was associated with enhanced microglial activation, increased proinflammatory cytokine release, and retinal ganglion cell loss, whereas miR-93 overexpression suppressed STAT3 signaling, attenuated inflammation, and conferred neuroprotection.^45^ Consistent with these findings, our data demonstrate downregulation of miR-93 in the aged retina, accompanied by increased IBA-1 immunoreactivity, indicative of heightened microglial activation. Together, these observations suggest that age-associated loss of miR-93 may contribute to sustained microglial activation and chronic inflammation, thereby exacerbating retinal degeneration. Modulation of the miR-17/93 gene network may therefore represent a potential strategy to restore microglial homeostasis in age-related retinal decline.

Other notable age-specific miRNAs that were dysregulated could also contribute to age-related degeneration in the retina, including the well-documented miR-146-5p, which was shown to be upregulated. The increased expression of miR-146- 5p has been well recognized to increase with age in several other tissues throughout the body, including the skin,^67^ adipose tissue,^68^ whole-blood PBMCs, plasma, bone, cartilage,^69^ lungs,^70^ kidney, and even the retina.^71^ An upregulation of miR-146a-5p has been postulated to play an active role in suppressing inflammation, given its mRNA targets are actively involved in inflammation, particularly via NF-κB activation.^19^ NF-κB signaling is known to act as the master regulator of the innate immune response, where miR-146 actively binds to several mRNA transcripts involved in the canonical pathway,^72^ where it has been shown using a miR-146a^−/−^ animal model that aging becomes accelerated via the early activation of NF-κB.^73^ Thus, miR-146a-5p may be upregulated in 30-month-old animals to repress sustained levels of inflammation that normally persist in the retina at an advanced age. Future studies could look at the mRNA retinal transcriptome of older miR-146a^−/−^ knockout mice to see if its mRNA targets are further enriched at an advanced age in the absence of this miRNA. The therapeutic potential of miR-146 has been shown to rescue the cognitive decline associated with neurodegenerative conditions, such as Alzheimer disease,^74^ but very little has been investigated in the context of the retina, especially retinal degenerations. One study has shown that overexpression of miR-146 in retinal microvascular endothelial cells reduces levels of TLR4/NF-κB, further reducing levels of inflammation as a result.^75^ This could suggest that supplementation of this miR-146a-5p in the retina may be therapeutic and could be investigated using the previously established photo-oxidative damage model.

The molecular landscape of the aged retina was further investigated through the integration of RNA sequencing and total proteomic analysis. This revealed age-related dysregulation of key proteins involved in interferon and cytokine signaling, Stat signaling, and broader inflammatory and immune pathways. In our dataset, we observed a poor overall correlation between mRNA and corresponding protein levels, contrasting prior reports that suggest transcriptomic and proteomic changes should overlap.^52^ One likely contributing factor is the inherent limitation of total proteomic analyses, which have reduced sensitivity for detecting low-abundance or cell type–restricted proteins. This constraint may result in underrepresentation of proteins expressed at low levels or within specific retinal cell populations, thereby diminishing apparent transcript–protein overlap. Improved depth of proteomic coverage and the application of cell type–resolved approaches would be necessary to more accurately characterize the molecular relationships underlying age-associated alterations. Additionally, although the inclusion of a single sex may limit the generalizability of our findings and precludes assessment of potential sex-specific differences,^76^ it does not diminish the significance of uncovering these novel molecular alterations. Future studies may incorporate molecular analyses of aged male mice to determine whether similar regulatory patterns are observed across sexes. However, for the purposes of this study, mice were group-housed, and female mice were selected to minimize aggression and fighting that are more commonly observed in group-housed aged males, which can introduce stress-related confounders and increase variability in molecular outcomes.

It is important to acknowledge that biological sex represents a key variable, particularly in aging research, where age-related hormonal changes, including the decline in estrogen, may influence retinal physiology, immune responses, inflammatory signaling, and miRNA expression profiles.^77^^,^^78^ Fluctuations in hormonal signaling, even in advanced age, may modulate inflammatory signaling and miRNA expression,^76^ potentially contributing to sex-specific regulatory networks. While this study was not designed to evaluate such differences, the use of a single sex reduced variability related to intersex comparisons and allowed for a more controlled characterization of age-associated molecular changes. While the lack of estrogen may exacerbate degeneration in models of neurodegeneration, comparable effects in the absence of overt retinal pathology appear less pronounced, although the available evidence remains limited.^78^^,^^79^ Extended overrepresentation analyses further suggest that the molecular signatures observed reflect age-related signatures rather than molecular responses to hormonal fluctuations. Notwithstanding, future studies directly comparing male and female aged cohorts will be essential to determine the extent to which these findings could be sex-dependent.

It should nonetheless be emphasized that estrogen is a well-established modulator of retinal and central nervous system physiology, and its regulation of neuroinflammatory signaling is directly relevant to the inflammatory networks reported here.^80^ Estrogen receptors are functionally expressed in the retina, where receptor-selective signaling can protect retinal neurons,^81^ yet the same hormonal environment can amplify glial interleukin-1β and increase neuronal vulnerability,^82^ and female-specific sex hormones have been shown to worsen photoreceptor degeneration through inflammatory, pyroptotic, and endoplasmic reticulum stress pathways in diseased, though not overtly healthy, retinas.^79^ Because the aged retinas examined here already exhibited microgliosis and photoreceptor loss, a contribution of hormonal status cannot be formally excluded. Furthermore, the estrous cycle was not staged, and the cumulative effects of lifelong estrogen exposure and its age-related decline were not controlled, all of which are known to shape neural gene expression.^83^ Our single-sex design therefore reduces intersex variability but cannot disentangle hormonal from chronological aging; dedicated studies incorporating estrous cycle staging, hormonal manipulation, and both sexes will be required to resolve this.

By integrating transcriptomic, proteomic, and miRNA data from retinas at an advanced age, we identified previously unreported molecular associations between dysregulated miRNAs, their mRNA targets, and corresponding protein expression patterns. This comprehensive approach highlights the cascade of regulatory events that occur in the retina at an advanced age and lays the foundation for future studies targeting key miRNA–mRNA–protein interactions involved in inflammation and degeneration.

These findings reveal coordinated molecular changes that occur at an advanced retinal age, although the cross-sectional nature of our data precludes inference of causality. Given the substantial photoreceptor loss observed in aged retinas, some of the detected molecular alterations may reflect shifts in cellular composition rather than changes in gene or protein expression within surviving cells. Although it was beyond the scope of this study to examine cell-specific changes, future studies employing cell type–specific approaches, such as single-cell transcriptomics^60^^,^^84^ or spatially resolved proteomics,^85^ will be essential to disentangle these effects and to more precisely define the cellular origins of age-related molecular signatures. A limitation of our study is that it compares young animals with very old animals and therefore captures molecular features of late-stage aging rather than the earliest initiating events. However, our enrichment analyses against independent aging datasets indicate that several of the transcriptional changes identified here are also present in younger aged cohorts, including comparisons such as 3 versus 20 months, supporting the relevance of these signatures across the aging trajectory. Future studies should include both male and female cohorts to determine the extent to which these findings are sex dependent. This will be particularly important given evidence from human disease that males may show increased susceptibility to retinal degenerative conditions such as age-related macular degeneration,^77^ which share inflammatory features with those identified in our study.

Finally, an inherent limitation of this study is that young and aged cohorts were maintained at separate facilities prior to experimental standardization. Although both groups consisted of female C57BL/6J wild-type mice housed under identical conditions with an acclimatization period before tissue collection, lifelong environmental differences, including diet^86^ and housing conditions, cannot be fully excluded and may influence immune and metabolic states. Nevertheless, here we report molecular changes using a well-characterized inbred, genetically stable animal line, and the consistent molecular and phenotypic patterns observed within each age group support age as the primary driver of the reported effects. Future longitudinal studies would further strengthen causal attribution to aging.

## Conclusions

These findings identify molecular targets associated with an advanced retinal age, particularly within inflammatory pathways, and reveal correlations between transcriptomic and proteomic dysregulation and age-related retinal degeneration. Our data highlight novel associations involving several miRNAs and their predicted molecular networks, providing new insight into the transcriptional landscape of the older retina.

## Supplementary Material

Supplement 1

Supplement 2
