# Supplementary material for: Multiomic Integration Reveals Novel miRNA–mRNA–Protein Expression Profile in the Aged Female Retina
Source: Invest Ophthalmol Vis Sci. 2026 Sep 17;67(11):37. doi: 10.1167/iovs.67.11.37 (PMC13602155; doi:10.1167/iovs.67.11.37)
Supplement: Supplementary file 1 [file iovs-67-11-37_s001.docx]

**Supplementary Figure 1: Bioanalyzer and read normalization for mRNA and miRNA samples**

(A) RNA quality was measured using the Bioanalyser 2100. (B) Power analysis for sample sizes and bulk mRNA sequencing normalization (C) Power analysis for sample size and miRNA sequencing normalization.

**Supplementary Figure 2: Optical Coherence Tomography**

Optical coherence tomography was used to quantify the thickness of retinal layers between young and 30 m mice. 30 m mice presented with significantly thinner ONL, INL, GCL+IPL and Whole Retina (WR) compared to the young control group.

**Supplementary Figure 3: Proteomic Quality Control**

(A) Total protein counts were calculated for each sample post-normalization to assess overall protein detection depth and consistency. (B) Protein intensity values were normalized across all samples to reduce technical variability and ensure comparability. (C) The proportion of missing values per sample was quantified to evaluate data completeness prior to imputation. (D) Missing protein intensity values were imputed using a low-intensity distribution-based approach to simulate values below the detection limit. (E) Power analysis for sample sizes n=6 for young, n=5 for aged.

**Supplementary Figure 4: Integration of mRNA sequencing and total proteomic data**

(A) Volcano plot showing 41 differentially expressed proteins. Principal Components Analysis (B) shows a higher variability and a shift to the left in the 30 m group compared to 3 m controls. (C) Biological enrichment using Gene Ontology reveals enrichment in inflammatory processes, as shown by STRING analysis (D), which indicates significant interactions with STAT signaling. (E) The integration of proteomic and RNA sequencing data revealed only 15 genes to be shared between the two datasets. (F) STRING analysis on these molecular targets that were enriched at both mRNA and protein levels are involved in immune response and inflammatory pathways.

**Supplementary Figure 5. No significant enrichment of cell death-related pathways identified in transcriptomic datasets.**
Biological enrichment analysis of differentially expressed transcripts against known cell death-related pathways revealed no significant enrichment within Gene Ontology Biological Processes or KEGG pathway databases in upregulated (A) or downregulated (B) transcripts. (C) Visualization of genes associated with these cell death pathways demonstrates a lack of coordinated or significant transcriptional enrichment.
